# Microfluidic synthesis of protein-loaded nanogels in a coaxial flow reactor using a design of experiments approach†

**DOI:** 10.1039/d0na01051k

**Published:** 2021-02-18

**Authors:** Zoe Whiteley, Hei Ming Kenneth Ho, Yee Xin Gan, Luca Panariello, Georgios Gkogkos, Asterios Gavriilidis, Duncan Q. M. Craig

**Affiliations:** School of Pharmacy, University College London 29-39 Brunswick Square London WC1N 1AX UK duncan.craig@ucl.ac.uk; Department of Chemical Engineering, University College London Torrington Place WC1E 7JE UK

## Abstract

Ionic gelation is commonly used to generate nanogels but often results in poor control over size and polydispersity. In this work we present a novel approach to the continuous manufacture of protein-loaded chitosan nanogels using microfluidics whereby we demonstrate high control and uniformity of the product characteristics. Specifically, a coaxial flow reactor (CFR) was employed to control the synthesis of the nanogels, comprising an inner microcapillary of internal diameter (ID) 0.595 mm and a larger outer glass tube of ID 1.6 mm. The CFR successfully facilitated the ionic gelation process *via* chitosan and lysozyme flowing through the inner microcapillary, while cross-linkers sodium tripolyphosphate (TPP) and 1-ethyl-2-(3-dimethylaminopropyl)-carbodiimide (EDC) flowed through the larger outer tube. In conjunction with the CFR, a four-factor three-level face-centered central composite design (CCD) was used to ascertain the relationship between various factors involved in nanogel production and their responses. Specifically, four factors including chitosan concentration, TPP concentration, flow ratio and lysozyme concentration were investigated for their effects on three responses (size, polydispersity index (PDI) and encapsulation efficiency (% EE)). A desirability function was applied to identify the optimum parameters to formulate nanogels in the CFR with ideal characteristics. Nanogels prepared using the optimal parameters were successfully produced in the nanoparticle range at 84 ± 4 nm, showing a high encapsulation efficiency of 94.6 ± 2.9% and a high monodispersity of 0.26 ± 0.01. The lysis activity of the protein lysozyme was significantly enhanced in the nanogels at 157.6% in comparison to lysozyme alone. Overall, the study has demonstrated that the CFR is a viable method for the synthesis of functional nanogels containing bioactive molecules.

## Abstract 2

A coaxial flow reactor is used to develop lysozyme-loaded nanogels, optimised *via* a design of experiments approach.

## Graphical abstract

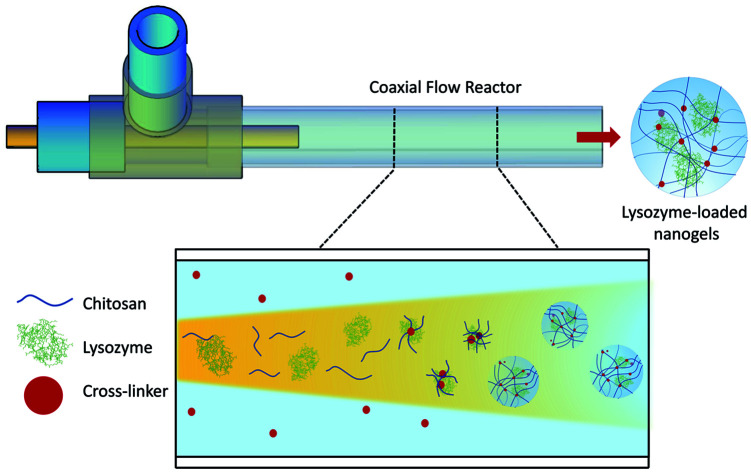

## 1. Introduction

The use of microfluidics for the manufacture of drug delivery systems is at a relatively early stage yet is arguably showing great promise.^1–4^ The term refers to the manipulation of fluid flow at a microscopic scale, allowing flow characteristics to be finely controlled for both rapid and homogeneous mixing. Microfluidics was first employed as an approach to microanalysis but rapidly became recognized as a versatile means of both analysis and production.^5^ Indeed, the miniaturisation of the manufacturing process enables portability, efficiency of processing with reduced reagent volumes, low variation between batches and the potential for scale-up where formulations may be optimised at low volumes and the process subsequently scaled up through parallelisation and increased device density.^6^ Advantages such as continuous manufacturing and low instrumental footprints can be achieved at the same time as improving the speed of reactions, increasing throughput and enhancing reproducibility of formulations,^7^ making the technology extremely attractive to the pharmaceutical industry.

Nanogels are described as dispersions of hydrogel nanoparticles based on crosslinked polymeric networks^8^ and their properties render them suitable for numerous drug delivery applications. Nanogels share many advantages with other nanocarrier systems such as aiding in the passage across biological membranes, large surface areas, potential for modification and derivatisation, avoidance of rapid renal clearance and increasing circulation half-life. Nanogels may also confer several other intrinsic advantages which render them attractive drug delivery candidates. Given their hydrophilicity, they can encapsulate a range of hydrophilic molecules, and their surrounding polymer network provides loaded therapeutics with a protective environment against degradation.^9^ Nanogels have a high capacity to hold water and an ability to swell in aqueous media, thus allowing imbibition of large quantities of water whilst preserving the internal cross-linked polymeric matrix.^10^ Often formed under mild aqueous conditions, these systems are ideal for the encapsulation of many biological molecules such as genes, proteins and peptides.^11^ Natural polymers are appealing for the formulation of nanogels given their biodegradability and biocompatibility.^12^ Chitosan is the natural polymer of choice in this study as its cationic nature allows complexation of its amine groups to negatively charged cross-linkers such as sodium tripolyphosphate (TPP) by ionic gelation.

At present, techniques relating to the synthesis of nanogels focus largely around ionic gelation alongside self-assembly and emulsification. Ionic gelation is one of the most widely used methods for fabricating nanogels, and refers to the use of polyelectrolytes forming cross-links in the presence of ions.^13^ Although simple and rapid, this technique is typically difficult to control given the instant assembly of polymer and cross-linkers, though certain factors have been identified to control the process such as; mass ratio of polymer to cross linker, temperature, charge density of cross-linker and polymer and also the pH.^14^ Ionic gelation is usually a batch process resulting therefore in batch to batch variations in physiochemical properties such as particle size, polydispersity, surface charge and drug release profiles,^15–17^ which is unfavourable for nanomedicines requiring a high level of quality control. There is therefore a compelling argument to consider the use of microfluidic approaches as an alternative and continuous means of producing nanogels of uniform size and architecture.

Microfluidic approaches have indeed been previously explored for this application, including a study by Bazban-Shotorbani *et al.*^18^ where alginate nanogels were formed by ionic gelation using hydrodynamic flow-focussing microchips to cross-link a core stream of calcium chloride and a sheath stream of alginate. This work was followed by Mahmoudi *et al.*^19^ who also synthesised alginate nanogels on microchips, where fine-tuning of the flow rate ratio allowed control over the size of nanogels produced. Huang *et al.*^20^ used the microchip platform to ensure controlled mixing and fabrication of hyaluronic acid based nanogels *via* photo-click cross-linking. Majedi *et al.*^21^ produced hydrophobically modified chitosan nanoparticles *via* self-assembly for the encapsulation of hydrophobic anticancer agents, where a T-shaped microfluidic chip was used to achieve particle synthesis. Efforts to produce chitosan/TPP nanogels using microfluidic have more recently been explored, where for example Chiesa *et al.*^22^ successfully formulated chitosan nanoparticles using a staggered herringbone micromixer in order to address the lack of reproducibility *via* batch production methods. In the formation of chitosan/ATP nanoparticles, Pessoa *et al.*^23^ observed channel obstruction in the microchips, whereby they implemented a central aqueous stream to allow for a large diffusion length for molecular diffusion, thus mitigating the fouling issues. Here we introduce the use of a coaxial flow reactor (CFR), whereby a core and sheath flow can be controlled by adjusting the flow rates and flow ratios accordingly. One of the main potential advantages of using a CFR in comparison to a microchip is that the event fouling may be greatly reduced; in the CFR the core solution is surrounded completely by the sheath flow and hence the reaction interface is shielded from the channel walls,^24^ giving a 3D flow-focussing effect. To our knowledge, this approach has not yet been implemented for the formation of nanogels and more specifically for chitosan/TPP nanogels. Rhee *et al.*^25^ compared the use of both 2D and 3D flow focussing devices for the production of polymeric nanoparticles and found that the 3D flow-focussing device consistently produced nanoparticles of smaller size and higher monodispersity which may be attributed to the greater efficiency of mixing. CFRs are also relatively simple to assemble, inexpensive and offer the ability to easily change the outer tube or inner capillary should any fouling occur, whereas serious fouling in a microchip often requires the fabrication of a completely new device. The use of CFRs in the literature is sparse and relates mainly to the synthesis of nanoparticles from silver,^24,26^ titania,^27^ iron oxide^28^ and polymeric nanoparticles,^3,4,25,29^ though the potential of such reactors and indeed microfluidics in general has yet to be explored fully in the pharmaceutical arena. Many of the current microfluidic devices used in the fabrication of pharmaceutical nanoparticles and nanogels are made from polydimethylsiloxane (PDMS)^2,30^ which usually swells in contact with organic solvents, resulting in the potential adsorption of small molecules into the channel walls.^4^ Further deposition of polymer on the channel wall may result in changes to the device structure and consequent negative effects on the mixing efficiency and robustness of nanoparticle production inside the device.

In the field of nanofabrication, it is not uncommon to find that several experimental factors can influence the properties of the resulting particles. Design of Experiments (DoE) is a technique which can be implemented to identify variables having a significant effect on response properties.^31^ In particular, response surface methodology (RSM) is able to identify important interactions between factors which are typically overlooked by ‘one variable at a time’ (OVAT) approaches.^32^ Using RSM requires fewer experiments to be carried out than a full factorial model and is capable of determining more effects than in OVAT approaches, including the interactions between factors and quadratic effects. Therefore, significant conclusions can be drawn whilst minimising cost and limiting wastage of reagents.

In the present study, DoE is used to optimise the nanogel fabrication process, reduce the number of experiments conducted and identify the optimal experimental conditions. The CFR is designed to add control to the fabrication of nanogels allowing regulation over particle size, polydispersity index (PDI) and encapsulation efficiency (% EE). Using the CFR, a core solution of chitosan and lysozyme is fed through an inner microcapillary and a solution of sodium triphosphate (TPP) and 1-ethyl-2-(3-dimethylaminopropyl)-carbodiimide (EDC) cross-linkers comprise the sheath flow through the outer glass tube, creating a 3D flow-focussing profile. TPP was used to form nanogels by ionic gelation, and the EDC is subsequently used to affect covalent cross-links in the nanogels.^33^ A four-factor three-level face-centered central composite design (CCD) was used to run a series of 27 experiments varying the chitosan, TPP and lysozyme concentrations and also the flow ratio, which is a ratio between the flow rates of the core and sheath solutions. The CFR set-up and the implementation of a dilute outer sheath stream allows us to mitigate the effect of fouling highlighted by Pessoa *et al.*^23^ which is a fundamental obstacle in enabling microfluidics to be widely used in formulating chitosan/TPP nanogels. The study aims to yield particles of a size below 100 nm, with a PDI below or equal to 0.3 and an % EE as close to 100% as possible, and so a desirability function was applied where each response was transformed into an individual desirability function in order to find the optimum conditions for successful nanogel production.

## 2. Methods

### 2.1. Materials

Medium molecular weight chitosan, TPP, EDC, lysozyme from chicken egg white (∼70 000 U mg^−1^) and *Micrococcus lysodeikticus* ATCC no. 4698 were purchased from Sigma-Aldrich (St. Louis, MO, USA). Glacial acetic acid and sodium hydroxide were of analytical grade and obtained from Fisher Scientific (Waltham, MA, USA). Ultrapure water was obtained from PURELAB® Chorus 2+ machine (ELGA LabWater, High Wycombe, UK). All solutions were filtered prior to entering the CFR through a 0.22 μm syringe filter unit from Merck Millipore (Billerica, MA, USA). Pierce bicinchoninic acid (BCA) protein assay kit was purchased from Thermo Fisher (Waltham, MA, USA). Amicon Ultra Diafiltration tubes (0.5 mL), with molecular weight cut-off of 30 kDa, were purchased from Merck Millipore (Billerica, MA, USA). The 0.595 mm ID glass microcapillary used to fabricate the CFR was purchased from Cole-Parmer (St Neots, UK), the 1.6 mm ID glass tube was purchased from VWR International Ltd (Lutterworth, UK). Polyether ether alkene (PEEK) T-junctions and perfluoroalkoxy alkane (PFA) tubing were purchased from Gilson Scientific (Middleton, WI, USA), while nuts and ferrules were purchased from Fisher Scientific (Waltham, MA, USA).

### 2.2. Fabrication of coaxial flow reactor

**Fig. 1:**
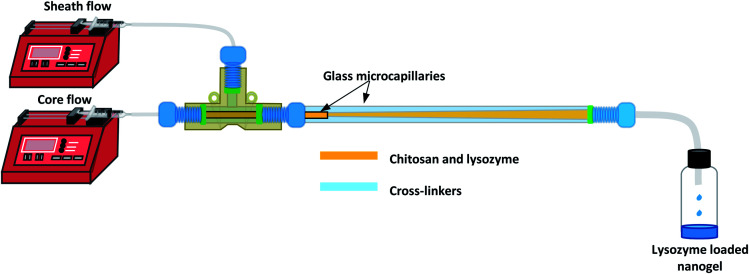
Schematic diagram of CFR experimental set up for lysozyme-loaded nanogel synthesis. One syringe pump delivers the core solution of chitosan and lysozyme at flow rates between 16 μL min^−1^ and 48 μL min^−1^ and another syringe pump controls the outer sheath solution of cross-linkers at a fixed flow rate of 160 μL min^−1^ to create flow ratios between 0.1 and 0.3. The diagram depicts a laminar flow system within the reactor, whereby the two fluid streams diffuse to allow mixing to occur.

The CFR as shown in Fig. 1 was designed in order to create a 3D flow-focussing profile. The reactor was assembled by fixing the smaller microcapillary of ID 0.595 mm and length 54 mm, into a larger glass tube with an ID of 1.6 mm and length 162 mm. The two tubes were fitted to a PEEK T-junction whereby chitosan and lysozyme of varying concentrations in accordance with the CCD were used as the core solution flowing through the inner microcapillary.

### 2.3. Experimental design

A systematic optimisation approach was employed in this study to determine the optimal nanogel fabrication parameters, with reference to three key parameters of nanogels including *Z*-average particle size (hydrodynamic particle size), PDI, and % EE. Based on our preliminary findings, four variables including chitosan concentration, TPP concentration, flow ratio and lysozyme concentration were considered important and were thus selected as the studied factors. We deliberately maintained the pH throughout the study at pH 5 in order to preserve the activity of the lysozyme and simultaneously dissolve the chitosan. A range of nanogels were prepared in the CFR by varying these factors in line with the DoE model.

#### 2.3.1. Central composite design

A face-centred central composite design (CCD) was used to study the effect of individual factors, the interactions and quadratic effects on nanogels. Three different levels were studied for each of the four factors and were codified in unitless values as shown in Table S1.† A total of 27 experimental runs, including 16 factorial points, 8 axial points and 3 centre points, were performed in triplicate. The experimental design matrix was created using JMP 15 (SAS Institute, Cary, NC, USA). Stepwise regression was used to fit the polynomial model to the data for each individual dependent variable and normal probability plots were performed to detect any outliers. A lack of fit test and a one-way analysis of variation (ANOVA) test were conducted to determine the accuracy of fit and statistical significance respectively for the model at a confidence interval (CI) of 95%. Response surfaces and contour plots were plotted using the same software to visualise the relationship between independent and dependent variables. A *p*-value of less than 0.05 is considered statistically significant.

#### 2.3.2. Optimisation and validation of experimental design

A multiple response optimisation was planned to determine the optimal conditions for lysozyme loaded nanogel fabrication, as the response variables may contradict one another. A desirability function, first proposed by Harrington *et al.*^34^ and later popularised by Derringer and Suich^35^ was implemented to transform each response variable (*y*_*i*_) into an individual desirability function (*d*_*i*_(*y*_*i*_)) with a scale between 0 and 1. *d*_*i*_(*y*_*i*_) = 0 indicates an undesirable response while *d*_*i*_(*y*_*i*_) = 1 represents an ideal or desirable response. The desirability functions were transformed using JMP 15 software by applying the criteria of minimising particle size and PDI and maximising % EE. The desirability was determined by the eqn (1) depending on the response, to either maximise or minimise the corresponding result.1
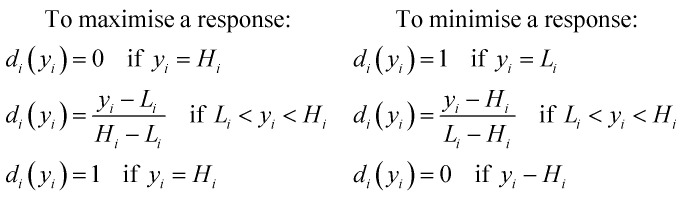
where *H*_*i*_ and *L*_*i*_ are the highest and lowest values for a response respectively, *d*_*i*_(*y*_*i*_) represents the individual desirability function of the factor concerned.

When a product or a process has multiple quality characteristics, simultaneous consideration of all responses is important to determine the optimal conditions of the independent variables. Thus, an overall desirability (*D*) was calculated, as the geometric mean of the combined individual desirabilities, as shown in eqn (2), where all responses are weighted equally. The operating condition with highest overall desirability was determined as the optimal conditions for nanogel fabrication in the CFR, which was identified *via* JMP 15.2
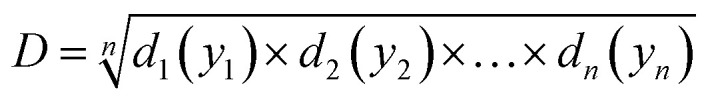
where *d*_1_(*y*_1_) and *d*_2_(*y*_2_) are the individual desirability functions for factor 1 and 2 respectively. With the total number of factors denoted as *n*, the individual desirability function for the factor *n* is therefore named as *d*_*n*_(*y*_*n*_).

A final experimental run was conducted in triplicate using this optimal condition to validate the experimental design. The predicted values of response variables obtained from the respective polynomial equations were compared to the experimental values with the percentage error calculated.

### 2.4. Synthesis of nanogels in CFR

The core and sheath solutions were prepared in accordance with the CCD matrix in Table 2. Chitosan solutions of concentrations between 0.1–0.3% (w/v) were prepared by dissolving chitosan in 1% (v/v) aqueous acetic acid solution and heating to approximately 60 °C under stirring until fully dissolved. Solutions were adjusted to pH 5 with diluted sodium hydroxide solution, to enable the amine groups of chitosan to be sufficiently positively charged, whilst also preserving the stability of lysozyme. Lysozyme was added to the chitosan solution to achieve final concentrations ranging from 0.1–0.3% (w/v), forming the core solution for the CFR. TPP was prepared in various concentrations between 0.01–0.02% (w/v) and EDC was added to this solution to obtain a fixed concentration of 0.5% (w/v) to allow its presence in excess and formed the sheath flow solution. The sheath flow was maintained at a constant flow rate of 160 μL min^−1^ and the core flow rate was varied to achieve differing flow ratios (core/sheath flow rate) between 0.1–0.3 to meet the DoE matrix requirements. A flow ratio of 0.3, for example, would represent a flow rate of 48 μL min^−1^ for the core solution and 160 μL min^−1^ for the sheath flow. All experiments were performed at ambient temperature.

**Table 1:** Experimental design of CCD showing the independent and dependent variables, as well as the measured zeta potential for each formulation (which was not included in the DoE model). The concentrations stated in the table are the initial concentration of solutions in the syringes. PDI is the polydispersity index and % EE is the encapsulation efficiency of the nanogels

Run	Independent variables				Dependent variables			Additional measurements (not included in DoE model)
	Chitosan conc. (% w/v)	TPP conc. (% w/v)	Flow ratio	Lysozyme conc. (% w/v)	Particle size (nm)	PDI	% EE	Zeta potential (mV)
1	0.3	0.010	0.3	0.1	63	0.818	91.2	16.0
2	0.1	0.020	0.3	0.3	113	0.199	88.1	20.8
3	0.2	0.015	0.3	0.2	103	0.295	97.1	23.1
4	0.1	0.010	0.3	0.1	105	0.249	94.3	23.0
5	0.2	0.015	0.2	0.2	117	0.205	95.5	21.4
6	0.1	0.015	0.2	0.2	118	0.203	72.6	21.7
7	0.2	0.015	0.2	0.1	131	0.213	79.1	23.1
8	0.2	0.020	0.2	0.2	123	0.251	83.1	21.8
9	0.3	0.010	0.3	0.3	46	0.675	96.5	16.2
10	0.1	0.020	0.1	0.3	1567^a^	0.474	31.3	21.3
11	0.1	0.010	0.1	0.3	129	0.19	62.4	18.3
12	0.3	0.020	0.3	0.1	168	0.198	91.0	23.4
13	0.3	0.015	0.2	0.2	216	0.163	93.5	23.2
14	0.3	0.020	0.3	0.3	147	0.248	96.8	16.7
15	0.2	0.015	0.1	0.2	134	0.203	80.1	21.1
16	0.3	0.010	0.1	0.1	100	0.21	75.9	21.3
17	0.1	0.010	0.3	0.3	64	0.299	96.6	19.8
18	0.1	0.010	0.1	0.1	188	0.257	17.2	20.9
19	0.3	0.020	0.1	0.1	238	0.368	54.3	20.2
20	0.1	0.020	0.1	0.1	5237^a^	0.425	23.9	20.1
21	0.1	0.020	0.3	0.1	133	0.209	83.8	24.2
22	0.2	0.010	0.2	0.2	77	0.29	95.5	21.0
23	0.2	0.015	0.2	0.3	105	0.223	95.0	22.6
24	0.3	0.020	0.1	0.3	125	0.222	86.9	17.3
25	0.2	0.015	0.2	0.2	111	0.199	95.4	22.2
26	0.2	0.015	0.2	0.2	99	0.223	95.6	22.0
27	0.3	0.010	0.1	0.3	221	0.154	91.8	23.4

a Excluded in the CCD model.

**Table 2:** Results of analysis of variance (ANOVA) and lack of fit tests for the individual response surface models of *Z*-average particle size, PDI and encapsulation efficiency of the lysozyme-loaded chitosan nanogels. The table shows the *F* value which may be used to determine the ratio of variance to unexplained variance, the prob. > *F* is the probability that the regression coefficients are zero and *R*^2^ demonstrates how well the model is fitted to the data, whilst the adjusted *R*^2^ (adj *R*^2^) is adjusted to the number of factors in the model

Model	Source of variation	Degrees of freedom	Sum of squares	Mean square	*F* value	Prob. > *F*	Significance	*R* ^2^ (adj *R*^2^)
*Z*-Average particle size	Model	4	27 540	6886	4.87	0.0066	Significant	0.493
	Residual	20	28 280	1413				(0.392)
	Lack of fit	9	10 820	1202	0.76	0.6560	Not significant	
	Pure error	11	17 450	1587				
PDI	Model	7	0.54	0.077	19.9	<0.001	Significant	0.880
	Residual	19	0.07	0.0039				(0.836)
	Lack of fit	7	0.04	0.0064	2.65	0.0660	Not significant	
	Pure error	12	0.03	0.0024				
% EE	Model	5	10 860	2172	16.0	<0.001	Significant	0.792
	Residual	21	2856	136.0				(0.792)
	Lack of fit	9	1893	210.3	2.62	0.0612	Not significant	
	Pure error	12	962.9	80.24				

### 2.5. Synthesis of nanogels by stirring approach

The optimal nanogel formulation as identified in the DoE model was also produced in batch by a conventional stirring method for nanogel production. Nanogels were made in 3 mL batches where the volumes of the TPP/EDC solution and the chitosan/lysozyme solution used were 2.3 mL and 0.7 mL respectively, in order to replicate the volume ratio used in the CFR when the flow ratio is 0.3. The TPP/EDC solution was added to a glass vial and placed under stirring at 600 rpm. The second solution of chitosan and lysozyme at pH 5 was added to the glass vial dropwise over a period of 39 seconds to mimic the mixing time in the CFR at the flow ratio of 0.3. The mixing in the vial was allowed to continue for 14.4 minutes to simulate the time that would be required for collection of the same volume (3 mL) in the microfluidic reactor.

### 2.6. Characterisation of lysozyme-loaded nanogels

#### 2.6.1. Dynamic light scattering (DLS) and capillary electrophoresis

The *Z*-average particle size and PDI of the nanogels were measured using a ZetaSizer Ultra (Malvern Panalytical, Malvern, United Kingdom) at room temperature. A disposable polystyrene cuvette was used in the analysis. Zeta potentials were measured using U-shaped capillary cells (DTS 1070, Malvern Panalytical, Malvern, United Kingdom). The results were obtained from three independent experiments, with triplicate measurements performed and results presented as the mean value ± standard deviation.

#### 2.6.2. Encapsulation efficiency and drug loading of lysozyme-loaded nanogels

For the encapsulation efficiency, lysozyme-loaded nanogel suspensions were loaded into a 0.5 mL Amicon Ultra Diafiltration tube (MWCO 30 000). The solutions were then centrifuged at a rotational speed of 14 000 × *g* for 30 minutes at room temperature using a mini centrifuge (SciSpin Micro Centrifuge, SciQuip, Wem, UK). The unencapsulated lysozyme in the nanogel suspension was determined by bicinchoninic acid (BCA) assay and % EE was calculated using eqn (3). The experiment was performed in triplicate and the results presented as mean value ± standard deviation.3
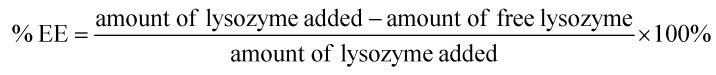
where amount of lysozyme added refers to the amount of lysozyme initially added into the formulation while amount of free lysozyme refers to the amount of lysozyme not encapsulated in the nanogels.

For the drug loading (% DL), the nanogels produced using the optimum formulation were freeze-dried for a duration of 48 hours. A sample of freeze-dried nanogels was taken from each replicate and a 2 mg mL^−1^ suspension obtained. The concentration of lysozyme in the nanogels was measured using a BCA assay and % DL was calculated using eqn (4).4
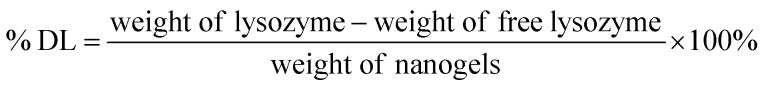
where weight of lysozyme refers to the weight of lysozyme calculated as from the BCA assay while weight of free lysozyme refers to the lysozyme that was not encapsulated in the nanogels as calculated previously. The weight of nanogels refers to the weight of the freeze-dried nanogels.

#### 2.6.3. Fourier transform infrared spectroscopy

The infrared spectra of raw materials and freeze-dried nanogels were obtained using a Spectrum 100 FTIR spectrometer with attenuated total reflectance (ATR) sampling accessory (PerkinElmer, Waltham, MA, USA) in the range of 650–4000 cm^−1^ and with a resolution of 1 cm^−1^.

#### 2.6.4. Transmission electron microscope

The shape and morphology of the nanogels obtained under the optimal conditions were characterised using a Philips/FEI CM120 BioTwin Transmission Electron Microscope (TEM) (FEI, The Netherlands), where one drop of the nanogel sample was placed on a 200-mesh carbon-coated copper grid and stained with 1% uranyl acetate solution, followed by air-drying at room temperature for a few minutes. Excess solution was removed using filter paper. The particle size distribution was determined on the TEM images using ImageJ (US NIH, Bethesda, MD, USA) with a sample size of *n* = 107 particles and a Gaussian fit was applied to the histograms. Two separate TEM images were used to achieve a sufficient number of measurements for the particle size analysis.

#### 2.6.5. Lysozyme activity assay

The activity of the lysozyme was determined by the rate of lysis of the bacteria *Micrococcus lysodeikticus*^36^ and the experiment was adapted from the protocol reported by Xie *et al.*^37^ 2.5 mL of 0.15 mg mL^−1^*Micrococcus lysodeikticus* cells suspended in phosphate buffer solution were pipetted into a clear-bottom 24-well plate and 0.1 mL of the samples to be tested were added to the 24-well plate including unloaded nanogels, lysozyme loaded nanogels and solutions of all raw materials used. A lysozyme solution with the equivalent concentration to the nanogel suspension of 0.3% (w/v) was added as a control to the 24-well plate. The 24-well plate was assayed in a plate reader (SpectraMax M2e; Molecular Devices, San Jose, CA, USA) at 450 nm. The change in absorbance was measured over 5 minutes, with absorbance recordings taken at 1 minute intervals. The maximum linear rate of change in absorbance was calculated and the bioactivity of lysozyme in nanogels was deduced from eqn (5). Statistical analysis of the lysozyme activity was carried out using analysis of variance (ANOVA) on GraphPad Prism software. Tukey's test was used to determine the significant difference between samples and the controls, where significance was defined as *p* < 0.05.5
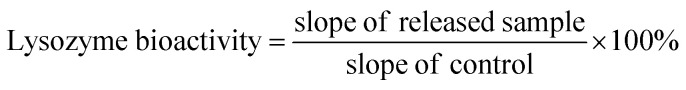
where slope denotes the rate of change of absorbance at 450 nm in the measuring samples or control *i.e.*, lysozyme only.

#### 2.6.6. Viscosity measurements

Dynamic viscosities of the core and sheath solutions were measured in order to calculate the mixing time in the CFR. Measurements were carried out with an automated rolling ball micro-viscometer (AMVN, Anton Paar, Graz, Austria), with 5 replicates measured at inclination angles of 60° and at 25 °C. A 1.6 mm glass capillary with 1.5 mm stainless steel balls were used for the measurement. Approximately 1 mL of sample was placed into the capillary.

## 3. Results and discussion

### 3.1. CCD development

Using the experimental design matrix, twenty-seven formulations of nanogels were prepared with the corresponding independent and dependent variables presented in Table 1. Responses were fitted with a polynomial model, which includes individual independent terms, their interaction and quadratic terms. The polynomial equation is comprised of coefficients *β*_0_ to *β*_14_ and the terms, as shown in eqn (6). Factors estimated to have an effect were selected by the stepwise regression for each individual response. If the lack of fit test was significant, terms with the highest *p*-value were further excluded from the model until the lack of fit test became insignificant. Two outliers were detected according to Cook's distances (data not shown) in the model for *Z*-average particle size, and thus these points were removed from the training set.

6 *Y* = *β*_0_ + *β*_1_*A* + *β*_2_*B* + *β*_3_*C* + *β*_4_*D* + *β*_5_*AB* + *β*_6_*AC* + *β*_7_*AD* + *β*_8_*BC* + *β*_9_*BD* + *β*_10_*CD* + *β*_11_*A*^2^ + *β*_12_*B*^2^ + *β*_13_*C*^2^ + *β*_14_*D*^2^

where *Y* represents the response, *A* represents chitosan concentration, *B* represents TPP concentration, *C* is the flow ratio and *D* is the lysozyme concentration.

#### 3.1.1. Statistical analysis

ANOVA and lack of fit tests were performed on the response surface model for each individual dependent variable, and the results reported in Table 2. ANOVA was performed to determine the correlation between the model and the responses, with the null hypothesis of no predictive capacity in these models. The *p*-values obtained were <0.05, which demonstrated that the quadratic models have capacity in predicting the responses. The lack of fit test for all models revealed that the lack of fit was not significant relative to the pure error. The square of the correlation coefficient (*R*^2^) and the root mean square error (RMSE) were also calculated to determine how well the model fitted with the experimental data and the predictive performance respectively. The *R*^2^ values were >0.75 for the models of PDI and % EE, which indicated that the models show a good fit to the experimental data and the responses are appropriately described by the models. However, the model for *Z*-average particle size did not fit as closely to the experimental data, with the *R*^2^ value of 0.49. The RMSE was calculated as 37.6, 0.062, and 11.7 for the models of *Z*-average particle size, PDI and % EE. The normalised RMSE were also converted and were reported as 29.6%, 21.9% and 14.5% for the models of *Z*-average particle size, PDI and % EE respectively.

#### 3.1.2. Multiple response optimisation

The optimal running conditions were determined by multiple response optimisation, with the aim of minimising the *Z*-average particle size and ensuring this is below 200 nm, minimising PDI and maximising the % EE, where all terms are weighted equally. The optimal running conditions for nanogel production were found to be 0.154% (w/v) chitosan concentration, 0.015% (w/v) TPP concentration, flow ratio at 0.3 and 0.3% (w/v) lysozyme concentration, as shown in Table 3. The predicted *Z*-average particle size, PDI and % EE of nanogels produced at the optimal conditions were 84.5 nm, 0.193 and 99.1% respectively. The overall desirability for the optimal condition was 0.85 as calculated from the desirability calculation in eqn (2) taking into account the individual desirability functions for each response.

**Table 3:** Optimum nanogel formulation results and a comparison between predicted and experimental values for each response variable. Optimum relates to nanogels produced with characteristics as close to the desired characteristics as possible, *i.e.*, *Z*-average particle size below 200 nm and as small as possible, PDI as close to 0 as possible and % EE as close to 100% as possible. Zeta potential of the optimum formulation was measured at pH 5.09 ± 0.03. The experimental values are calculated based on three separate batches of nanogels, where three measurements were taken for each batch and an overall standard deviation calculated to measure the variance between batches

Factor	Optimal value	Response	Predicted value	Experimental value	Mean difference (%)
Chitosan conc.% (w/v)	0.154	Particle size (nm)	84.5	84.0 ± 4.0	0.6
TPP conc.% (w/v)	0.015	PDI	0.193	0.261 ± 0.007	35.2
Flow ratio	0.3	EE (%)	99.1	94.6 ± 2.9	4.5
Lysozyme conc.% (w/v)	0.3	Zeta potential (mV)	N/A	19.9 ± 0.6	N/A
		DL (%)	N/A	73.1 ± 1.8	N/A

To validate the accuracy of the model in predicting the optimal conditions, an additional experimental run was carried out at the optimal running conditions. The experimental results measured were 84.0 ± 4 nm (*Z*-average particle size), 0.261 ± 0.007 (PDI), 94.6 ± 2.9% (% EE) as reported in Table 3 and the profile in Fig. S1.† A good correlation between the predicted and experimental responses indicated that the models for *Z*-average particle size and % EE have sufficient accuracy in predicting the optimal condition. However, the larger mean difference between the measured and predicted PDI may indicate that other factors not included in this model might also affect the PDI of the nanogels.

The percentage drug loading was performed on the optimum formulation of nanogels and found to be 73.1% ± 1.8% (w/w). Nanogels have repeatedly demonstrated their ability to accommodate a high drug loading efficiency, often achieved by physical entrapment of drugs^38^ or in the case of biological agents, high loading is attained by electrostatic, van-der Waals and/or hydrophobic interactions with the polymeric network interactions.^39^ Hydrophilic nanogels typically show a high drug loading capacity given that in their unloaded state they hold a high-water capacity, which provides sufficient space for large quantities of biological agents to be loaded.^39^

**Fig. 2:**
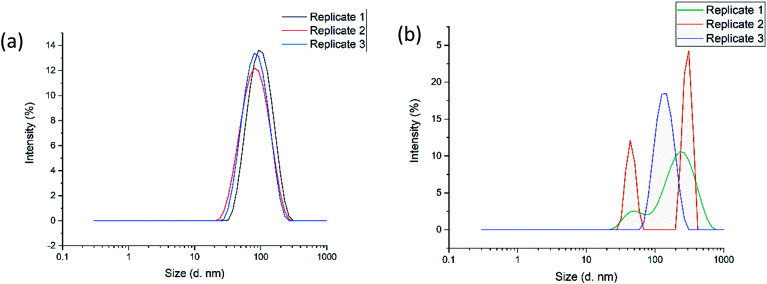
(a) Graph showing particle size and distribution of the optimal formulation of nanogels produced by microfluidics in the CFR and (b) shows the particle size and distribution of the optimal formulation of nanogels produced by a conventional stirring approach for comparison. Each graph depicts the results of three replicates of nanogels produced by each method.

The optimal formulation for the nanogels as determined by the DoE model, was carried out both in the CFR and also by a conventional stirring approach for comparison. It is evident from Fig. 2(a) that nanogels produced in the CFR are composed of only one size population, as only one single peak was observed for each of the three replicates tested. In Fig. 2(b) the three replicates tested for nanogels produced by a conventional stirring approach have multiple populations of sizes given the various peaks that can be seen. This highlights that the CFR is a reproducible method for formulating the nanogels in this study, whereas the stirring approach in batch yields high levels of size variation. The average size of the nanogels produced in the CFR was 84.0 ± 4.0 nm with a PDI of 0.261 ± 0.007, whereby the batch produced nanogels had a size of 319.1 ± 307.5 nm with a high PDI of 0.492 ± 0.446. Such results indicate that the stirring approach in batch is less able to control the particle size and reduce the polydispersity of the nanogels in this study. The large variation between the three replicates, as indicated by the standard deviation, also shows that the formulation is not reproducible when using this approach.

### 3.2. Effects of factors on responses

The model terms were selected in stepwise regression, wherein the terms were chosen based on the estimated effect only. However, not all of the effects for these selected terms were statistically significant at the 95% confidence interval (*p* < 0.05) and at that particular degree of freedom despite the ANOVA result for the overall model being statistically significant. Therefore, the effect of the individual, interaction and quadratic terms selected in the CCD models were also evaluated and are reported in Table 4. The interaction of chitosan concentration and lysozyme concentration (*AD*), and the interaction between TPP concentration and lysozyme concentration (*BD*), quadratic effects of flow ratio (*C*^2^) and lysozyme concentration (*D*^2^) were not found to affect any of the properties of nanogels and thus are not represented in Table 4.

**Table 4:** Effects of the individual factors, interaction and quadratic effects on the dependent variables – *Z*-average particle size, PDI and % EE in their corresponding CCD models. The factors correspond to those defined in eqn (6)

Factors	*Z*-Average particle size			PDI			EE%		
	Sum of squares	*F*-Ratio	*p*-Value > *F*	Sum of squares	*F*-Ratio	*p*-Value > *F*	Sum of squares	*F*-Ratio	*p*-Value > *F*
*A*	331.350	0.234	0.6336	0.017	4.351	0.0507	2394.089	17.61	0.0004^b^
*B*	9102.017	6.438	0.0196^a^	0.017	4.303	0.0519			
*C*	15 703.230	11.107	0.0033^a^	0.026	6.763	0.0176^a^	5394.835	39.67	<0.001^b^
*D*							1009.053	7.42	0.0127^a^
*AB*				0.080	20.694	0.0002^b^			
*AC*				0.118	30.479	<0.001^b^	1626.711	11.96	0.0024^b^
*BC*				0.217	56.072	<0.001^b^			
*CD*							435.453	3.20	0.0880
*A* ^2^	5510.756	3.898	0.0623						
*B* ^2^				0.066	16.918	0.0006^b^			

a *p*-Value < 0.05.

b *p*-Value < 0.01.

#### 3.2.1. Mixing time in the CFR

The mixing time in the CFR can be evaluated from eqn (7):7
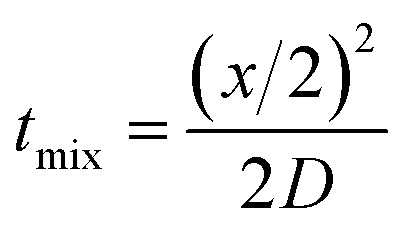
where *x* is the diffusion distance and *D* represents the diffusion coefficient. *x* can be estimated as a function of the flow ratio from the work of Abou-Hassan *et al.*,^40^ who report the value of the core stream thickness as:8
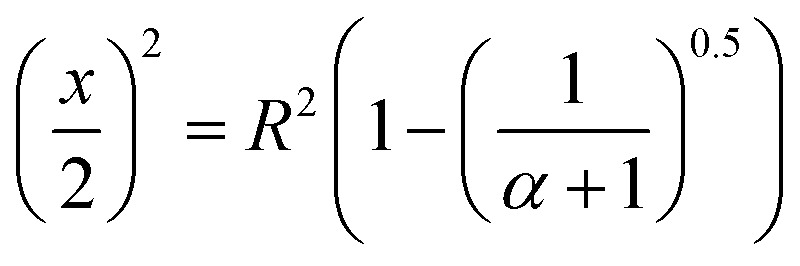
where *R* is the outer tube radius and *α* is the ratio between inner and outer flow rates. Using this approach, one calculates a mixing time ranging from 15 to 39 s, when increasing the flow ratio from 0.1 to 0.3. The overall average residence time in the reactor, given by *τ* = *V*/(*Q*_in_ + *Q*_out_), ranges between 110 and 94 s when varying the flow ratio between 0.1 and 0.3. Note that the value of *x* is computed assuming that the flow is fully developed, hence not accounting for the increase in the sheath stream radius along the developing length, and viscosity differences between the two streams were not considered.

The mixing times have been computed using a typical diffusion coefficient of 10^−9^ m^2^ s^−1^ for TPP in water. This is a significant assumption, as the chitosan inner stream is more viscous than water, hence one could argue that the diffusion coefficient is lower. The viscosity of the chitosan solutions was found to be approximately two times larger than water (see Table S2†). Using the highest viscosity (*i.e.*, 2.8 cP) and considering that the diffusivity is inversely proportional to the viscosity, we would get a diffusivity coefficient of 3.6 × 10^−10^ m^2^ s^−1^, resulting in a range of mixing time between 42 and 109 s. Considering the uncertainties in the value of the diffusion coefficient of TPP in water and the assumptions of the equations above, these calculations suggest that the ratio *t*_mix_/*τ* ∼ 1. Given this result, it could be argued that the reaction may not reach completion inside the flow rector. One would expect then that this would be cause of irreproducibility and poor particle size distribution, as sample collection at the reactor outlet is a rather uncontrolled process, which however does not appear to be the case.

#### 3.2.2. Effect of factors on particle size

Controlling the size of nanogels is of crucial importance due to the effects on permeability, cellular uptake, half-life and drug release.^41^ In this study the ideal particle size was around 100 nm and definitely below 200 nm. Nanoparticulate systems of size below 10 nm are typically cleared quickly by renal filtration and are not reabsorbed, whilst nanocarriers between the sizes of 50–200 nm are often unable to escape from continuous blood capillaries.^42^ Fabricating nanogels of sufficiently small size is difficult to achieve in conventional nanogel fabrication methods given the rapid and uncontrolled mixing involved in their formulation. Of the twenty-seven DoE experiments performed in this study, only five of those yielded particles of size greater than 200 nm, demonstrating that the CFR can successfully formulate nanogels of a desirable size.

**Fig. 3:**
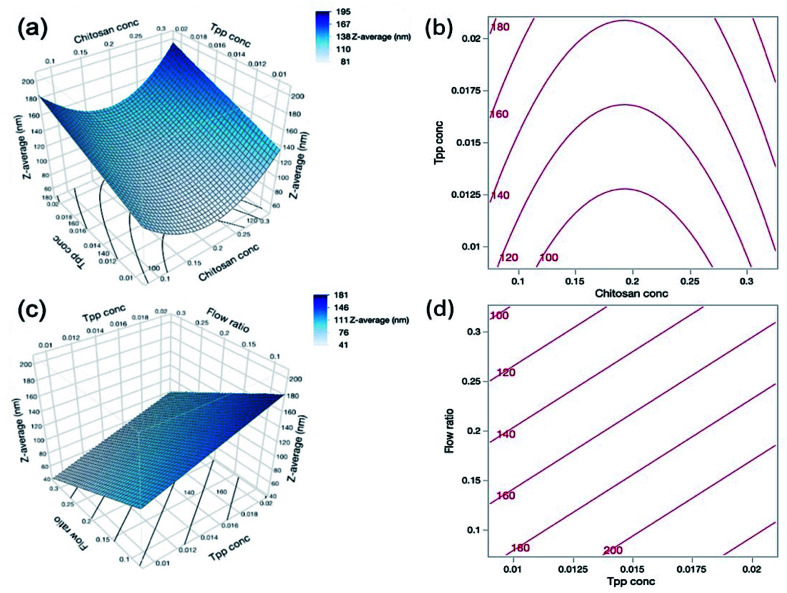
(a) Response surface and (b) contour plot showing the predicted interaction effects of chitosan concentration and TPP concentration on the *Z*-average particle size of nanogels. (c) Response surface and (d) contour plot showing the predicted interaction effects of TPP concentration and flow ratio. The response surface and contour plots are only able to compare 2 process factors at once, and so where parameters are not included in the interaction but had a statistically significant effect on the particle size, their midpoint value was used. For example, in (a) the flow ratio is 0.2 and for (b) the chitosan concentration is 0.2% (w/v).

As can be seen in Table 4, both the TPP concentration and flow ratio were shown to be statistically significant factors influencing the nanogel size. TPP is the anionic cross-linker of choice in this study and is able to complex with the cationic charge of chitosan. Eqn (9) shows the positive effect of TPP concentration (*B*) on nanogel size, and so as the TPP concentration increases, so too does the particle size; we suggest that this is because the increased concentration allows for additional cross-links to form within the nanogel structure, thus forming larger particles. Fig. 3(c and d) depict the effect of both TPP concentration and flow ratio on the particle size.

9 Size (nm) = 217.7222 − 1205.0000*A* + 4926.6667*B* − 323.5556*C* + 3130.0000*A*^2^

According to eqn (9), the flow ratio (*C*) had a negative value, indicating that an increase in flow ratio results in a decrease in the particle size. At higher flow ratios the flow rate of the core solution is increased whilst the sheath flow remains constant, and so more chitosan and lysozyme are added to the formulation. Therefore, the additional positive charge of the chitosan and lysozyme may allow further charge interaction and condensation of the nanogel particles resulting in the decreased size.

Furthermore, as the flow ratio increases the ratio of chitosan and TPP is also increased, which has been shown in the literature to vastly affect the particle size. Koukaras *et al.*^43^ observed that the particle size decreased to a minimum of 340 nm at a chitosan/TPP ratio of 4/1 and increased thereafter. This is in agreement with Zhang *et al.*^44^ who achieved a decrease in particle size with increasing ratio until 5/1 where particles were at a minimum of 109 ± 4 nm, and particles increased in size thereafter. This indicates that although in this work the chitosan concentration was statistically insignificant on the particle size, the flow ratio was significant and therefore there may be an optimum flow ratio for the formation of nanogels in the same way that there is an optimum chitosan/TPP ratio when using standard mixing techniques. The effect of chitosan concentration and TPP concentration on the particle size is depicted in Fig. 3(a and b). It is interesting to note, that despite increases in the chitosan concentration with increasing flow ratios, there was no fouling in the reactor and at the tip of the inner microcapillary throughout all experiments.

#### 3.2.3. Effect of factors on PDI

The term polydispersity index (PDI) refers to the size distribution around the mean particle size. PDI values range between 0–1, where the larger values correspond to a broader size distribution and may indicate a high degree of aggregation within the sample. In drug delivery systems where a highly monodisperse sample is desirable to ensure consistency in drug loading, % EE, drug release and cellular uptake, a value of 0.3 or below is generally considered as acceptable,^45^ a value of 0.7 or above would indicate that the particles have an extremely broad size distribution. The CFR used in this study offers the potential to formulate nanogels with a high degree of monodispersity, which is a common challenge faced in batch production techniques. The regression model describing the relationship between factors and PDI is shown in eqn (10) below:

10 PDI = 0.3284 + 0.7117*A* − 56.6056*B* + 2.1598*C* − 141.6250*AB* + 8.5938*AC* − 233.1250*BC* + 4182.2222*B*^2^

**Fig. 4:**
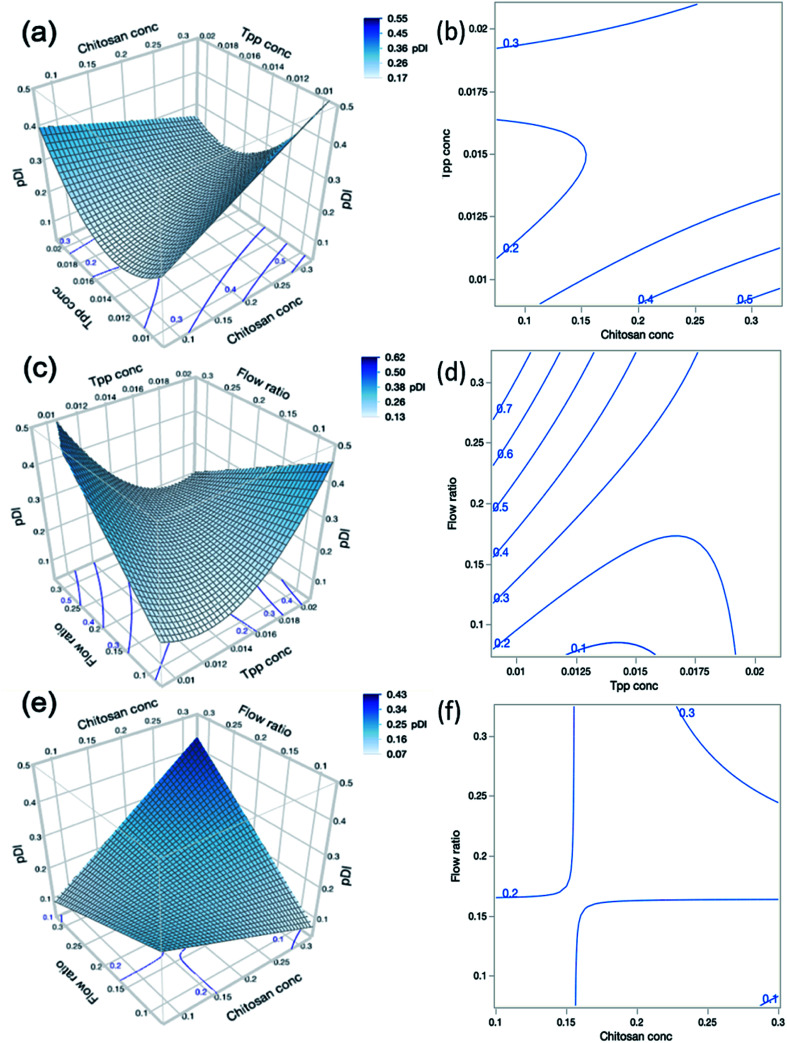
(a) Response surface and (b) contour plot showing the predicted interaction effects of chitosan concentration and TPP concentration on the PDI of the nanogels. (c) Response surface and (d) contour plot showing the predicted interaction effects of TPP concentration and flow ratio. (e) Response surface and (f) contour plot showing the predicted interaction effects of chitosan concentration and flow ratio on the PDI of nanogels. The response surface and contour plots are only able to compare 2 process factors at once, and so where parameters are not included in the interaction but had a statistically significant effect on the particle size, their midpoint value was used. For example, in (a) the flow ratio is 0.2, in (b) the TPP concentration is 0.015% (w/v) and in (c) the chitosan concentration is 0.2% (w/v).

As shown in Table 1, the CFR successfully formulated nanogels with a PDI below 0.3 for 22 out of 27 experimental runs, with PDI values reaching as low as 0.154. Where the highest PDI values of 0.818 for run 1 and 0.675 for run 9 were obtained, both of these formulations also had the lowest zeta potential at +16.0 and +16.2 mV respectively. This may indicate that the particles are more likely to aggregate given the insufficient charge repulsion between nanoparticles leading to a higher propensity for aggregation. Both run 1 and run 9 also had the lowest level of TPP present in the formulation at 0.01% (w/v) and the highest level of chitosan at 0.3% (w/v) which may infer that there was an insufficient amount of cross-linker to allow adequate particle formation to occur. Such findings are also confirmed by the results presented in Table 4 which show that the PDI was significantly influenced by the interaction between chitosan concentration (*A*) and TPP concentration (*B*) where this interaction had a *p*-value less than 0.0002. This is demonstrated in contour plots shown in Fig. 4(a), which shows that at lower TPP concentrations PDI increases with increasing chitosan concentrations. In contrast, at higher TPP concentrations, an increase in the concentration of chitosan led to lower PDI values. It is suggested that as more chitosan becomes available to form cross-links with TPP, nanogels with greater uniformity in size distribution could be obtained as the chitosan–TPP ratio has been optimised. It is possible that optimum parameters for the size and PDI of nanogels using the CFR system differs, as can be observed from the difference in the optimum for size and PDI in Fig. 3(a) and 4(a) respectively. The overall desirability calculation is therefore used to determine a balance between optimum parameters required for size, PDI and % EE.

Additionally, only flow ratio (*C*) amongst all individual factors exhibited a statistically significant effect on the PDI (*p* < 0.05) as shown in Table 4. An increased flow ratio signifies a higher flow rate of the core solution whilst the sheath flow rate remained constant. This therefore causes an increase in the overall flow rate and an increase in the mixing time, as discussed previously in Section 3.2.1. The increased mixing time with higher flow ratios causes an increase in PDI, which may indicate that the residence time is not high enough to accommodate the requirement for increased mixing and thus the mixing may be incomplete at higher flow ratios.

As shown in Fig. 4(c), there is an interaction effect of chitosan concentration and flow ratio, whereby at the low concentrations of chitosan, increasing the flow ratio leads to a decrease in PDI. This is due to the fact that at low chitosan concentration and low flow ratio simultaneously, there is an insufficient amount of chitosan in the system to adequately cross-link particles hence leading to larger variations in particle sizes and high PDI values. However, as the flow ratio increases, a sufficient amount of chitosan is introduced into the system to cross-link with TPP and thus the PDI reduces. The cross-interactions between chitosan concentration, TPP concentration and flow ratio (*AB*, *AC* and *BC*) also exhibited statistically significant effects on the PDI of the nanogels (*p* < 0.0001).

It should also be noted that the quadratic effect of TPP concentration (*B*^2^) was also statistically significant on PDI (*p* = 0.0006), indicating that as TPP concentration increases, PDI decreases until a minimum point before increasing again. This again indicates that there is an optimum concentration of TPP required for the nanogel formation. If the TPP level is too low there is an insufficient amount to form adequate cross-linking in the nanogels, and when the level is too high this may introduce aggregation due to inter-particular cross-links.

#### 3.2.4. Effect of process factors on encapsulation efficiency

Another property rendering nanogels ideal drug delivery candidates is their ability to encapsulate a range of therapeutic molecules.^41,42,45^ Microfluidics is also employed in this study to enhance the % EE, given that the mixing of the therapeutic agent is improved and distributed more evenly throughout the particles, thus promoting a high degree of encapsulation. Positive coefficients were observed in eqn (11) for linear effects of chitosan concentration and flow ratio, indicating that the % EE increased with increasing chitosan concentration and flow ratio. By both increasing the chitosan concentration and flow ratio there is an increased level of polymer in the formulation which allows a high amount of protein to be retained and encapsulated in the particles.

**Fig. 5:**
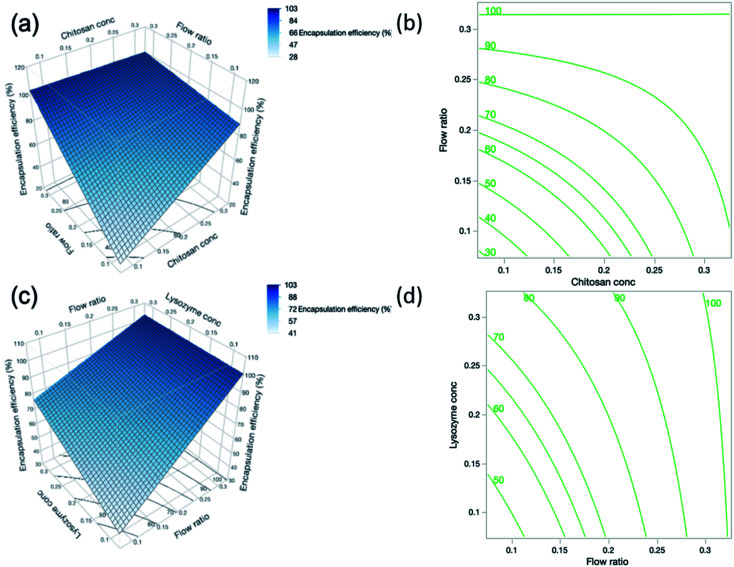
(a) Response surface and (b) contour plot showing the predicted interaction effects of chitosan concentration and flow ratio on the encapsulation efficiency of the nanogels. (c) Response surface and (d) contour plot showing the predicted interaction effects of flow ratio and lysozyme concentration. The response surface and contour plots are only able to compare 2 process factors at once, and so where parameters are not included in the interaction but had a statistically significant effect on the particle size, their midpoint value was used. For example, in (a) the lysozyme concentration is 0.2% (w/v) and in (b) the chitosan concentration is 0.2% (w/v).

The interaction between chitosan concentration and flow ratio on % EE was also statistically significant (*p* = 0.0024). These findings are reflected in the surface profile and contour plot in Fig. 5(a and b) respectively, showing that the % EE increased with chitosan concentration and flow ratio, and then levelled off at high chitosan concentration and flow ratio. This observation is similar to that reported by Deng *et al.*^46^ who found an initial increase in % EE. However, this holds true until a certain concentration of chitosan, beyond which % EE decreased. When the concentration of chitosan further increases, the greater viscosity of the polymer may hinder the diffusion of lysozyme into the nanoparticle system in order to be encapsulated.

The concentration of lysozyme also had a strong influence on % EE and the positive relationship is indicated by the sign of the coefficient in eqn (11). As lysozyme concentration increases, % EE increases. This trend was demonstrated in DoE runs 19 and 24, where the smallest lysozyme concentration of 0.1% (w/v) (run 19) yielded a % EE of 54.3% and increasing lysozyme concentration to 0.3% (w/v) (run 24) gave a higher % EE of 86.9% despite otherwise having identical formulations. By increasing the lysozyme concentration, more nucleation seeds become available for nanogel growth to occur and hence there may be a higher number of particles which are able to encapsulate more lysozyme. In contrast, the interaction effect between flow ratio and lysozyme concentration shown in Fig. 5(c and d) was deemed statistically insignificant according to the statistical analysis.

11 EE% = −53.6944 + 316.9903*A* + 479.1222*C* + 179.2097*D* − 1008.3125*AC* − 521.6875*CD*

### 3.3. Fourier-transform infrared spectroscopy

**Fig. 6:**
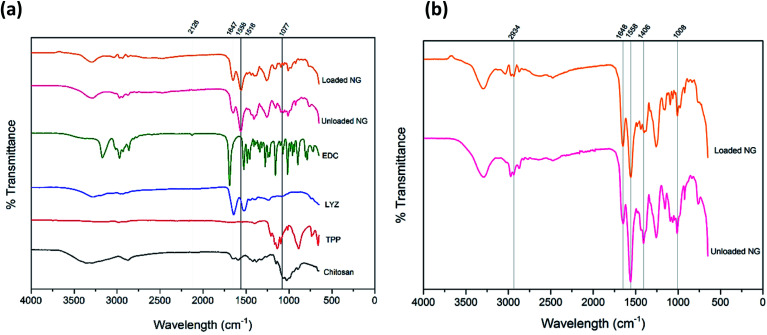
(a) FTIR spectrum showing all components of the formulation individually, unloaded nanogels and the optimum formulation of lysozyme loaded nanogels (b) FTIR spectrum showing the direct comparison of unloaded and loaded nanogels where the particle structure remains unchanged in the loaded and unloaded nanogels.

Fig. 6(a) shows the FTIR spectrum for both unloaded and loaded chitosan nanogels along with corresponding individual components of EDC, lysozyme, TPP and chitosan for reference, whilst Fig. 6(b) has been provided for a detailed comparison between the unloaded and loaded nanogels. The broad bands at 3295 cm^−1^ in the spectra of both loaded and unloaded nanogels can be attributed to the stretching vibration of N–H overlapped with the O–H stretching vibration.^47–49^ The C=O group from the amide structure of acetylated portions of chitosan can be seen at 1647 cm^−1^ in the nanogels and the second amide peak at 1558 cm^−1^ due to N–H bending and C–N stretching which was also observed by Wu *et al.*^50^ Given that such peaks are present in loaded and unloaded gels, may indicate that nanogels retain their structure upon incorporation of lysozyme.^50^ The peaks at 1406 cm^−1^ in the nanogel spectra are likely due to O–H bending in the structure of chitosan. The peak present in nanogels at 1008 cm^−1^ can be attributed to the C–O stretching of groups present in the ether group of chitosan and has shifted from 1077 cm^−1^ in the spectrum of chitosan alone. At 1258 cm^−1^ the sharp peak present in the nanogels is indicative of the P=O bond in TPP within the nanogel structure, albeit shifted from 1209 cm^−1^ in TPP alone as a result of the interaction with chitosan. The peaks at 1647 cm^−1^ and 1516 cm^−1^ for lysozyme can be attributed to the amide peaks I and II respectively.^51^

For the TPP spectra, findings were in agreement with those of Loutfy *et al.*^47^ where in our study, stretching vibrations can be seen at 1209 cm^−1^ assigned to the P=O bond, a peak around 1136 cm^−1^ attributed to the O–P=O group and a peak around 886 cm^−1^ belonging to the stretching vibration of the PO_3_ group. Such peaks can be seen in the nanogel formulations, though given the low quantity of TPP in the optimum formulation they are much weaker than in TPP alone. The peak at 2126 cm^−1^ in the EDC spectra is characteristic of the carbodiimide group (N=C=N), this is absent in both the unloaded and loaded nanogels. The absence of the carbodiimide group in the nanogel formulation is representative of the reaction of EDC and nitrogen whereby the absence of a carboxylic acid group in the chitosan is due to the direct reaction between the free amine groups of chitosan and the EDC, forming a covalent bond.^52^ A summary of the peaks and their attributions can be found in Table S3.† In conclusion, the FTIR confirms the presence of the individual components and the lack of structural change of the nanogels upon encapsulation and loading of lysozyme.

### 3.4. Shape and morphology of the nanogels

**Fig. 7:**
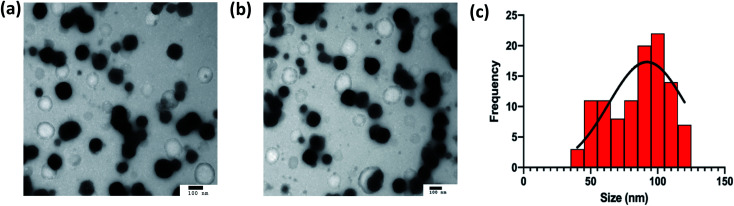
(a and b) TEM images of nanogels formulated using optimal conditions. The scale bars represent 100 nm (c) the histograms represent the distribution of size (*n* = 107) across the 2 TEM images shown.

The nanogels observed were largely spherical in shape, as observed in Fig. 7(a). The average diameter of the optimal formulation of nanogels is measured to be 92.0 ± 28.5 nm (*n* = 107) using ImageJ, which is in agreement with the value observed by DLS of 84.4 ± 4 nm albeit with a smaller standard deviation around the mean. The difference may arise from the fact that the size measured in the image was the diameter at the dry state, while the size measured in DLS was hydrodynamic size, of which particles were suspended in water with an assumption of the particles being spherical. The wide size distribution demonstrated on the TEM images may indicate that the drying stage required to prepare the TEM samples was not sufficient as some of the nanogels may not be fully dried and may appear larger than others. The drying of the nanogels can also allow aggregates to form, which may appear as larger particles on the TEM as observed in Fig. 7(a and b) thus contributing to the wide size distribution seen.

### 3.5. Lysozyme activity assay

**Fig. 8:**
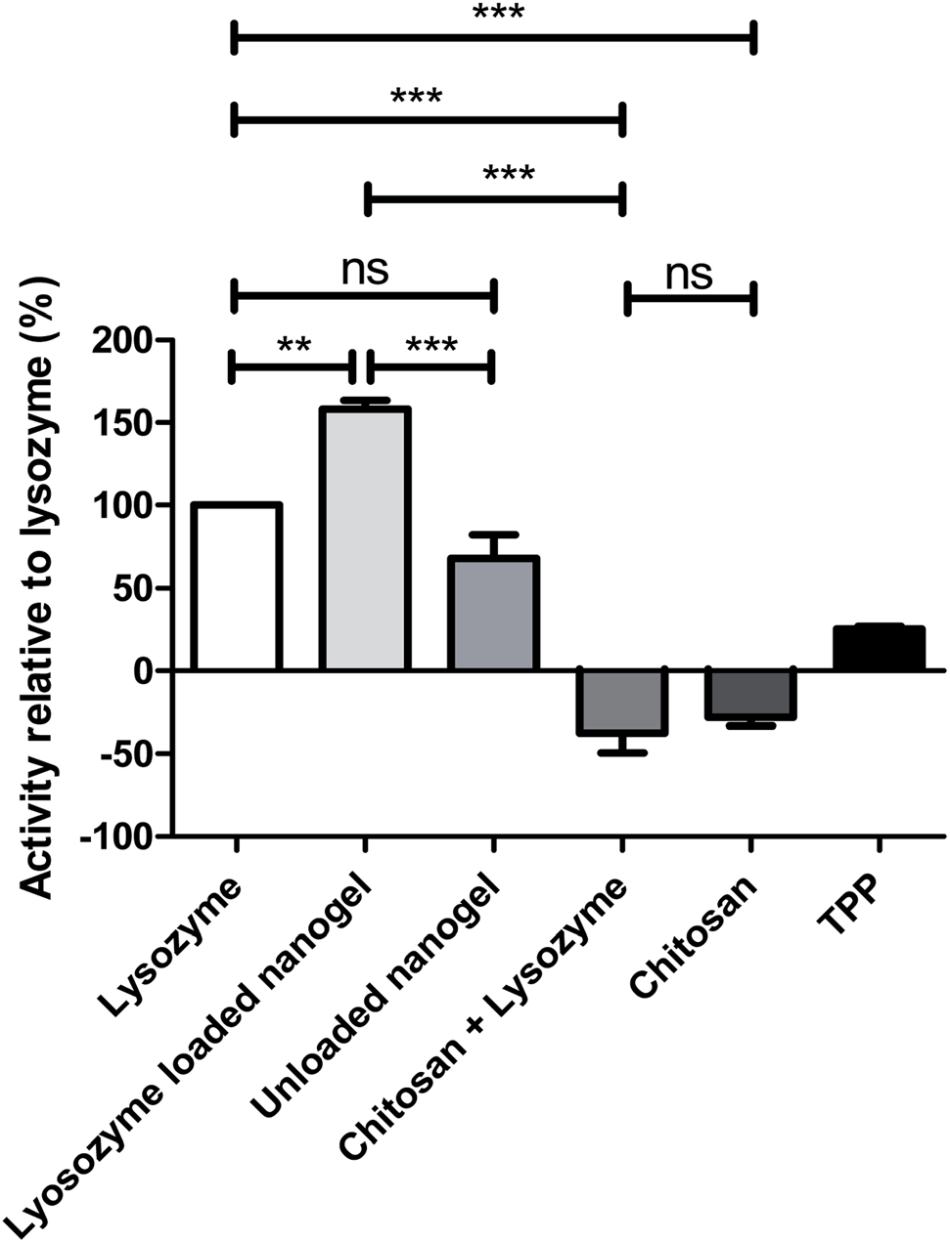
Activity of individual components of the formulation and the overall lysozyme loaded formulation against the cell wall of *Micrococcus lysodeikticus*. Analysis of variance (ANOVA) was performed to determine the significant difference in activity between the samples and the control, where Tukey's test was used to compare the difference in activity between samples. The first bar represents the lysozyme only control against which the other components are compared. **p-Value < 0.001, ***p-value < 0.0001. ns refers to *p*-value > 0.05. For the samples tested containing lysozyme, the concentration was maintained at 0.3% (w/v) consistent with the concentration in the optimum formulation.

The lysozyme activity assay was carried out on the optimum formulation in order to assess both the activity of the protein after processing in the CFR and encapsulation within nanogels. The rate of lysis of *Micrococcus lysodeikticus* cells was measured after exposure to native lysozyme compared to individual components of the nanogels and lysozyme loaded nanogels as can be seen in Fig. 8. Chitosan alone, and chitosan in combination with lysozyme exhibited negative activity values, indicating that bacterial growth continues and thus such components have little or no activity against the bacterial cell walls of *Micrococcus lysodeikticus* cells. Positive values were observed for TPP, unloaded nanogels and lysozyme loaded nanogels, indicating that these components are active on the lysis of the bacterial cell wall. Interestingly, individual components of the formulation have negligible effect in comparison to native lysozyme, whereas the unloaded nanogel and the loaded nanogels show an activity of 68.4% and 157.6% respectively. The activity of the lysozyme was therefore enhanced greatly in the nanogel formulation in comparison to lysozyme alone as the control represented at 100% in Fig. 8. The unloaded nanogels still exert an activity against *Micrococcus lysodeikticus* cells at 68.4% that of the lysozyme control. This effect can be explained in the study by Atay *et al.*^53^ who found that when chitosan is used in low concentrations, such as in the nanogels formulated in the CFR given the dilution from the sheath flow, the chitosan is able to bind to the negatively charged cell surface and disrupt the cell wall causing leakage of intracellular components and consequently rapid cell death. However, in the chitosan solution and chitosan and lysozyme combination where chitosan was present at higher concentrations, the chitosan may simply coat the cell surface and prevent leakage of intracellular components. The high encapsulation efficiency of the nanogels may also contribute to concentrating the lysozyme at the site of action thus allowing it to exert its activity on the bacterial cell wall.

## 4. Conclusions

In this study, chitosan nanogels loaded with the protein lysozyme were successfully synthesised using microfluidics in a coaxial flow reactor (CFR). Given that microfluidics is a somewhat new technique in the field of nanogels, a face-centered central composite design (CCD) was employed to determine the interaction of various factors involved in nanogel production and their responses. The four investigated factors were chitosan, lysozyme, tripolyphosphate (TPP) concentrations and flow ratio, and their effects on the particle size (*Z*-average particle size), polydispersity (PDI) and encapsulation efficiency (% EE) were established. Following the application of a desirability function to find a set of operating conditions which yield nanogels with desired characteristics, nanogels were produced with the optimum conditions (chitosan concentration 0.154% (w/v), TPP concentration 0.015% (w/v), flow ratio 0.3 and lysozyme concentration of 0.3% (w/v)). The predictability of the experimental model was subsequently determined by comparing the predicted and experimental values of all measured outcomes. The *Z*-average particle size of the optimum nanogel formulation was predicted to be 84.5 nm and the experimental value was 84.0 ± 4.0 nm, the PDI predicted to be 0.193 and the obtained value was 0.261 ± 0.007, and the % EE predicted to be 99.1% and an actual of 94.6 ± 2.9% was achieved. Overall, this shows that the Design of Experiments (DoE) model was able to achieve a high level of predictability for the properties of nanogels, and in combination with the CFR successfully enabled the production of nanosized, highly monodisperse nanogels with the ability to encapsulate a significant amount of lysozyme. Using response surface methodology (RSM), we were able to ascertain significant interactive effects between factors, in particular for PDI and % EE. The mixing time in the CFR was calculated, leading to the ratio between mixing time and residence time, *t*_mix_/*τ* ∼ 1. This would suggest that the CFR, operated in the conditions explored here, provides controlled and reproducible mixing, rather than fast mixing, enabling reproducible synthesis of chitosan nanogels. Furthermore, the use of this system prevents the lysozyme to experience high shear during the loading process, hence preserving its biological functions.

Importantly, a lack of fouling was observed throughout the study when using the CFR system, this being a common obstacle when using microfluidics with polymeric materials. This was achieved through the use of glass microcapillaries and a 3D flow-focussing system which is able to shield the reaction interface from the channel wall. The CFR enabled the core flow of polymer to be surrounded completely by the sheath flow of aqueous cross-linker solution to produce controlled mixing and consequently nanogels of high monodispersity, adequate size, and high encapsulation efficiency. The current ionic gelation technique for nanogel production lacks adequate control over product characteristics due to inefficient mixing and batch-to-batch variations. The CFR therefore offers a novel approach to formulating nanogels loaded with therapeutic agents, where mixing is controlled and homogeneous, products are reproducible between batches and scale-up in the pharmaceutical industry is possible. Although the use of microfluidics and particularly the CFR have not yet been extensively explored for drug delivery purposes, such techniques allow the fine tuning of parameters and ultimately production of nanogels with precise characteristics.

## Conflicts of interest

No conflict of interest to declare.

## Supplementary Material

NA-003-D0NA01051K-s001
