# Supplementary material for: Microfluidic synthesis of protein-loaded nanogels in a coaxial flow reactor using a design of experiments approach
Source: Nanoscale Adv. 2021 Feb 18;3(7):2039–55. doi: 10.1039/d0na01051k (PMC9419594; doi:10.1039/d0na01051k)
Supplement: NA-003-D0NA01051K-s001 [file NA-003-D0NA01051K-s001.pdf]

Electronic Supplementary Material (ESI) for Nanoscale Advances.
This journal is © The Royal Society of Chemistry 2021

Supplementary Information

Table S1

Non-codified and codified factor levels for the central composite design

	Non-Codified level	Low	Mid	High
	Codified level	-	0	+
**Factor**	**Symbol**			
Chitosan conc. (%w/v)	(A)	0.1	0.2	0.3
TPP conc. (%w/v)	(B)	0.010	0.015	0.020
Flow ratio (core/sheath flow rate)	(C)	0.1<br>(16 μLmin^{-1}/ 160 μLmin^{-1})	0.2<br>(32 μLmin^{-1}/ 160 μLmin^{-1})	0.3<br>(48 μLmin^{-1}/ 160 μLmin^{-1})
Lysozyme conc. (%w/v)	(D)	0.1	0.2	0.3

Table S2

Viscosities of the various solutions used in the DOE experimental model

Sample number	Chitosan (% w/v)	Lysozyme (% w/v)	Dynamic viscosity (cp)
1	0.1	0.1	1.4885
2	0.1	0.2	1.4907
3	0.1	0.3	1.4737
4	0.2	0.1	2.1568
5	0.2	0.2	2.1152
6	0.2	0.3	1.9188
7	0.3	0.1	2.8353
8	0.3	0.2	2.1215
9	0.3	0.3	2.0294

Table S3

Table showing functional groups and their corresponding wavelengths in the FTIR spectra shown in Fig.5 (a) and (b)

Wavelength (cm^{-1})	Functional Group
2126	Carbodiimide group (N=C=N)
1647	Amide group peak I (C=O)
1558-1516	Amide group peak II N-H bending and C-H stretching at 1558 cm^{-1} (shifted from 1516 cm^{-1} in lysozyme alone)
1406	O-H bending
1258 - 1209	P=O of TPP in nanogels at 1258cm^{-1} (shifted from 1209 cm^{-1} in TPP alone)
1136	O-P=O of TPP

1077 – 1008	C-O stretching of the ether group in chitosan at 1077cm^{-1} (shifting to 1008 cm^{-1} in the nanogels)
886	Stretching vibrations of PO_3 in TPP
